# The effect of Mediterranean Diet adherence on the relationship between cognitive performance and cancer survivorship

**DOI:** 10.1007/s11764-025-01879-1

**Published:** 2025-08-11

**Authors:** Andrew McLeod, Kelsey Gabel, Kaitlin Chakos, Jeffrey S. Wefel, Lisa Tussing-Humphreys, Vanessa M. Oddo

**Affiliations:** 1 Institute for Health Research and Policy, University of Illinois Chicago (UIC), Chicago, IL USA; 2 Department of Kinesiology and Nutrition, UIC, Chicago, IL USA; 3 Departments of Neuro-Oncology and Radiation Oncology, The University of Texas MD Anderson Cancer Center, Houston, TX USA

**Keywords:** Cognition, NHANES, Mediterranean Diet, Cancer

## Abstract

**Background/Objectives:**

Up to 75% of cancer survivors experience cognitive impairment from cancer or its treatment. No modifiable risk factor has been identified; however, a Mediterranean Diet may be protective. We sought to determine if the relationship between cognitive performance and cancer survivorship depends on Mediterranean Diet adherence.

**Methods:**

We used the latest data from the National Health and Nutrition Examination Survey (NHANES). Mediterranean Diet adherence was defined as the aMed score which gives 1 point for intake above/below the sample-specific median for healthful/unhealthful food groups (range 0–9). Cognitive processes assessed included processing speed, attention, working memory, learning, short-term memory, and executive function. Linear regression models adjusting for demographics, diet, and health behavior were used to determine the relationship between cognition and cancer history.

**Results:**

Among 2450 participants, 385 had cancer. Mean age was 68.9 years (SE = 0.2). The most common cancer was breast cancer (24%). Cancer history was not associated with cognition (*p* > 0.05). Among high aMed scores, cancer history was more negatively associated with number of Intrusions compared to low aMed scores (*β* (95% CI): 0.61 (0.07, 1.16), *p* = 0.03 for aMed*Cancer interaction term).

**Conclusions:**

Cancer history was more negatively associated with cognition among high versus low aMed scores. Because of the cross-sectional nature of NHANES, the limited number of cancer survivors, and the lack of treatment information, larger prospective studies with treatment data are needed.

**Implications for Cancer Survivors:**

Cancer survivors may require dietary recommendations different from that of the general population. More studies, however, are needed to confirm this.

## Introduction

Cognitive impairment associated with cancer or its treatment, called cancer-related cognitive impairment (CRCI), has been shown to impact a substantial proportion of cancer survivors [5, 31, 43]. For example, among breast cancer survivors (BCS), the prevalence of CRCI 3–5 years after chemotherapy is 21% [50]. Those with CRCI suffer from reduced quality of life and increased rates of stress, anxiety, depression, and post-traumatic stress disorder, compared to cancer survivors without CRCI [4, 21, 35]. Pharmacological and non-pharmacological treatments for CRCI are still actively being identified and tested, but improving diet quality is a plausible intervention that has received relatively little attention (PDQ [28],). Given that the number of persons in the United States (U.S.) with CRCI is expected to increase due to improving cancer survival rates [3], it is important to better understand whether the association between cancer survivorship and cognition varies by diet.

A recent systematic review of six studies found that fruit and vegetable consumption benefitted cognitive performance or reduced cognitive complaints among cancer survivors [10],however, prior literature on the association between diet and cognition in cancer survivors is limited. For one, prior studies typically investigated subjectively—versus objectively—measured cognition, which may be related more to stress [10, 23]. Additionally, the few studies investigating objective cognition and diet have a small sample size [30, 54] (*N* = 54–61). The sample size in Huang et al. [22] is 1047 breast cancer survivors, but there were no controls, precluding the ability to identify dietary patterns, specific food groups, or nutrients that may be more beneficial, or harmful, for survivors compared to controls [22].

To address the limitations of prior literature and to better inform dietary interventions for treating CRCI, we used the National Health and Nutrition Examination Survey (NHANES) to determine if past cancer history is associated with objectively measured cognition and to test whether that association varies by diet. Prior literature demonstrated greater objective cognitive deficit in cancer survivors compared to non-survivors in NHANES (1999–2002 cycles) [52]. Adherence to a Mediterranean Diet (Med Diet) has also been associated with higher cognitive scores in the majority of studies in cancer-free cohorts (van den [49]). Thus, we hypothesized that past cancer history would be associated with a cognitive deficit and that this negative association would be mitigated among those with a higher adherence to a Med Diet.

## Methods

### Overview

To test our hypotheses, we used NHANES data from 2011 to 2014. The design of NHANES has been published elsewhere [8, 24]. Briefly, NHANES is an ongoing cross-sectional study that collects data every 2 years using a clustered, probability sampling design whereby participants are randomly selected in a four-stage procedure with over-sampling of specific groups of public health concern. After providing informed consent, participants are administered several questionnaires at home. These questionnaires collect data about demographics, physical activity, medical conditions, and other domains. Further examination takes place at a Mobile Examination Center where a physical examination, cognitive evaluation, and a 24-h dietary recall are conducted. The physical exam includes blood work and measurement of height, weight, body composition, among others. The cognitive evaluation was administered to participants 60 years and older with no language or literacy requirements. No ethics approval was required as NHANES data is de-identified and publicly available.

### Participant selection

Participants in either the 2011–2012 or 2013–2014 waves of NHANES (*N* = 19,931) were initially included. We selected these waves because they were the most recently available waves that included the Consortium to Establish a Registry for Alzheimer’s Disease (CERAD) Word List test, Digit Symbol Substitution Test (DSST), and Animal Fluency, measures of verbal episodic memory, processing speed, and verbal fluency, which are frequently affected by cancer and cancer therapy [14, 44]. Individuals < 60 years of age were excluded as they were not eligible for cognitive assessment (*n* = 16,299). Additional exclusion criteria included the following: missing day 1 dietary recall (*n* = 528), missing total kilocalories (kcal) (*n* = 36), implausible kcal intake (< 500 or > 3500 kcal/day for women and < 800 or > 4200 kcal/day for men [48]) (*n* = 125), no saturated fat intake (a component for the Med Diet score, described below (*n* = 1)), missing one or more cognitive tests or one or more trials within any test (*n* = 335), unknown or missing cancer history (*n* = 3), or a history of non-melanoma skin or brain cancer (*n* = 154). Individuals remaining (*n* = 2450) constituted our sample for analysis.

### Independent variable: past cancer history

Past cancer history was determined with the following question: “Have you ever been told by a doctor or other health professional that you had cancer or a malignancy of any kind?” Participants could choose from 30 predefined cancer types.

### Dependent variables: cognitive performance

Cognitive performance included the following: (1) number correct on the DSST which assesses processing speed, sustained attention, and working memory; (2) number of words learned across three learning trials (CERAD-WL) and (3) a short delay (CERAD-DR), which assesses learning and short-term memory; (4) total intrusions on the CERAD; and (5) number correct on the Animal Fluency test which assesses language and executive function [6]. Higher scores represent better cognitive performance except for CERAD-Intrusions, where higher scores represent worse cognitive performance.

### Confounders, mediators, and effect modifier

Using a directed acyclic graph (DAG) [11], we identified plausible confounders as age (right censored at 80 years per NHANES design), race, ethnicity, household income, education, sex, body mass index (BMI), and smoking.

Variables deemed to be mediators were those on the causal path from cancer to cognition identified in the DAG. These included minutes of moderate to vigorous physical activity (MVPA) per week, sleep hours per night, depressive symptoms, and cardiometabolic disease. Physical activity and sleep decrease substantially post-cancer diagnosis, whereas rates of cardiometabolic disease and moderate to severe depressive symptoms increase significantly post-cancer diagnosis [15, 18, 20, 42, 45, 51, 53]. These conditions can impact cognition [12, 13, 34, 37, 46]. Cardiometabolic disease was captured under the variable comorbidity burden (range from 0 to 9) whereby a 1 was assigned if an individual had any of the following conditions: stroke, heart attack, angina, congestive heart failure, coronary heart disease, type 2 diabetes, high cholesterol, high blood pressure, or liver condition. Higher scores represent greater cardiometabolic disease burden.

Mediators were not included in models as this may have attenuated the total effect observed for the association between cancer survivorship and cognition.

In models testing the association between cancer survivorship and cognition, Med Diet adherence was included as an effect modifier. Adherence was defined by the alternate Mediterranean Diet score (aMED) [16], modified to be compatible with 24-h dietary recalls [17]. The score range is 0–9, with 1 point awarded for intake above the sex-specific median for vegetables, fruits, legumes, whole grains, nuts, seafood, and the ratio of monounsaturated fats to saturated fats, 1 point for intake at or below the sex-specific median for red meat and processed meats combined, and 1 point for no to moderate levels of alcohol consumption, i.e., 0–25 g of ethanol per day for women and 0–50 g of ethanol per day for men; and 0 points for any other level of intake for each of the 9 groups. Given the relatively small sample size compared to other studies (Molina-Montez et al., 2020), Med Diet adherence was dichotomized at the median, instead of being divided into smaller quantiles. High adherence was defined as a score > 4, and low adherence was defined as a score of 4 or less.

Additionally, we tested the extent to which the association between past cancer history and each measure of cognitive performance varied by age (< 75 or ≥ 75).

### Sensitivity analysis

We performed a sensitivity analysis to determine if survival bias may be influencing our results. By virtue of being long-term cancer survivors, such survivors may have had highly survivable and non-aggressive cancers and needed less aggressive treatment. Less aggressive cancer and treatment have been associated with less cognitive impairment [9, 40]. Long-term survivors may have also recovered from CRCI. Short-term survivors as a group, however, may have a greater proportion of individuals, compared to long-term survivors, with less treatable, more aggressive cancers requiring more aggressive treatment. Therefore, short-term survivors may have worse cognition compared to controls, whereas long-term survivors may not.

To determine if past cancer history’s effect on cognition varies by time since cancer diagnosis, we replaced the cancer term in our models with a categorical term coded as 0 = non-survivor, 1 = short-term survivor (< 5 years since diagnosis), and 2 = long-term survivor (≥ 5 years since diagnosis). We then investigated the strength and significance of this new categorical cancer term. Because age and age at diagnosis were right censored at 80 years, we could not accurately identify every survivor 80 years of age or older as a short-term or long-term survivor. Therefore, in this sensitivity analysis, we excluded anyone (including non-survivors) 80 years of age or older.

### Statistical analysis

We performed a descriptive analysis comparing cancer survivors to non-cancer controls on key variables (Table 1). We report means and standard errors for continuous variables and frequencies and percentages for categorical variables.

**Table 1:** Sample characteristics^A^

	Overall (*N* = 2450)		Cancer survivor (*n* = 385)		Control (*n* = 2065)		*p*^G^
	*N* (%) or mean (SE)		*N* (%) or mean (SE)		*N* (%) or mean (SE)		
*Age (years)*	68.9	0.2	70.8	0.4	68.5	0.2	< 0.01
*Sex*							0.38
Male	1176	44.8%	185	41.9%	991	45.4%	
Female	1274	55.2%	200	58.1%	1074	54.6%	
*Household income*							0.30
≥ $20,000	1746	83.1%	290	85.1%	1456	82.7%	
< $20,000	604	16.9%	80	14.9%	524	17.3%	
*Education*							0.01
< 9th	270	5.5%	35	4.1%	235	5.8%	
9th–12th, no diploma/GED	354	10.5%	39	6.2%	315	11.4%	
HS grad or GED	582	22.6%	86	21.6%	496	22.8%	
Some college or associates	684	31.0%	114	32.4%	570	30.7%	
≥ College grad	558	30.4%	111	35.6%	447	29.3%	
*Race/ethnicity*							0.01
Non-Hispanic White	1143	79.1%	223	84.9%	920	77.9%	
Hispanic	478	7.3%	52	5.0%	426	7.8%	
Non-Hispanic Black	608	8.8%	86	7.3%	522	9.1%	
Other	221	4.8%	24	2.8%	197	5.2%	
*Smoker*							0.04
Never	1208	50.2%	166	44.6%	1042	51.3%	
Former	930	39.1%	181	45.7%	749	37.8%	
Current	311	10.7%	38	9.8%	273	10.9%	
*aMed score (0–9)*	4.3	0.1	4.3	0.1	4.3	0.1	0.97
*BMI (kg/m*^*2*^*)*	29.3	0.2	29.3	0.4	29.2	0.3	0.92
*Comorbidity burden (0–8)*^*B*^	1.8	0.0	1.9	0.1	1.7	0.0	0.09
*Cancer type*^*C*^							
Breast	92	3.8%	92	23.9%	0	0.0%	
Prostate	88	3.6%	88	22.9%	0	0.0%	
Melanoma	40	1.6%	40	10.4%	0	0.0%	
Blood cancer^D^	16	0.7%	16	4.2%	0	0.0%	
Other cancer types^E^	149	6.1%	149	38.7%	0	0.0%	
Multiple cancers	36	1.5%	36	9.4%	0	0.0%	
Childhood cancer^F^	2	0.1%	2	0.5%	0	0.0%	
*Cognition*							
DSST Score	52.4	0.6	52.1	1.0	52.5	0.7	0.74
CERAD-WL	19.7	0.2	19.8	0.3	19.7	0.2	0.63
CERAD-DR	6.3	0.1	6.2	0.1	6.3	0.1	0.38
CERAD-Intrusions	0.6	0.0	0.6	0.1	0.6	0.0	0.54
Animal Fluency	18.1	0.2	18.1	0.4	18.1	0.2	0.85

^A^Ns are unweighted and percentages, means, and SEs are weighted using the survey weights

^B^Comorbidity burden is the number of comorbidities a subject has. Comorbidities include stroke, heart attack, angina, congestive heart failure, coronary heart disease, type 2 diabetes, high cholesterol, high blood pressure, and liver condition

^C^Not all cancer types are shown. Breast, prostate, and melanoma were the three most common cancers

^D^Blood cancer is defined as having a history of leukemia, blood cancer, or lymphoma/Hodgkin’s disease

^E^Other cancer types include: bladder, bone, cervical, colon, esophageal, gallbladder, kidney, larynx, liver, lung, mouth, nervous system, ovarian, pancreatic, rectal, soft tissue (muscle or fat), stomach, testicular, thyroid, and uterine

^F^Childhood cancer is defined as being diagnosed with cancer at less than 20 years of age

^G^*p* values were determined from the following regression models: ordinary least squares regression for normal, continuous variables (aMed score, BMI, comorbidity burden, DSST Score, CERAD-WL, CERAD-DR, Animal Fluency), censored linear regression (age, education), logistic regression (income), multinomial logistic regression (sex, race, smoking status), Poisson regression (CERAD-Intrusions)

We used linear regression models to test the association between cancer survivorship and each measure of cognitive performance (Table 2) and whether that association varied by aMed modeled as a continuous or binary variable (Table 3) and age (< 75 or ≥ 75) (Table 4). Model 1 was unadjusted. Model 2 adjusts for demographic characteristics only (education, sex, age, race, ethnicity, and income). Model 3 further adjusted for health and health behavior, i.e., diet-related variables (aMed score, kcals, day of recall, time of year of recall), BMI, and smoking.

**Table 2:** Regression estimates^A^ for modeling of cognition by past cancer history

	Cancer history *β* (95% CI)	*p*
DSST Score		
Model 1: Cancer history	− 0.42 (− 2.95, 2.12)	0.74
Model 2: Model 1 + Demographics^B^	− 0.47 (− 2.52, 1.58)	0.64
Model 3: Model 2 + Health and Health Behavior^C^	− 0.10 (− 2.01, 1.82)	0.91
CERAD-WL		
Model 1: Cancer history	0.10 (− 0.31, 0.51)	0.63
Model 2: Model 1 + Demographics^B^	0.27 (− 0.15, 0.69)	0.20
Model 3: Model 2 + Health and Health Behavior^C^	0.28 (− 0.14, 0.70)	0.19
CERAD-DR		
Model 1: Cancer history	− 0.12 (− 0.39, 0.15)	0.38
Model 2: Model 1 + Demographics^B^	− 0.01 (− 0.31, 0.28)	0.93
Model 3: Model 2 + Health and Health Behavior^C^	− 0.01 (− 0.30, 0.28)	0.93
CERAD-Intrusions		
Model 1: Cancer history	0.06 (− 0.14, 0.26)	0.54
Model 2: Model 1 + Demographics^B^	0.05 (− 0.16, 0.26)	0.63
Model 3: Model 2 + Health and Health Behavior^C^	0.06 (− 0.16, 0.27)	0.60
Animal Fluency Score		
Model 1: Cancer history	− 0.07 (− 0.75, 0.62)	0.85
Model 2: Model 1 + Demographics^B^	− 0.13 (− 0.70, 0.44)	0.65
Model 3: Model 2 + Health and Health Behavior^C^	− 0.062 (− 0.62, 0.50)	0.83

Shown are estimates of the coefficients for past cancer history for each of the five cognitive scores for each model used. Higher scores represent better cognitive performance except for CERAD-Intrusions, where higher scores represent worse cognitive performance

^A^Estimated using ordinary least squares regression, except for CERAD-Intrusions for which Poisson regression was used

^B^Demographics: age, race, gender, income, education

^C^Health and health behavior: diet, BMI, and smoking

**Table 3:** Association^A^ of past cancer history with cognition by Med Diet adherence

Model 3: Model 2^B^ + Health and Health Behavior^C^	Cancer history *β* (95% CI)	*p*	Cancer history*aMed *β* (95% CI)	*p*
DSST Score	2.0 (− 2.9, 6.9)	0.42	− 0.50 (− 1.52, 0.52)	0.32
CERAD-WL	0.87 (− 0.91, 2.64)	0.33	− 0.14 (− 0.53, 0.25)	0.47
CERAD-DR	0.29 (− 0.63, 1.20)	0.53	− 0.07 (− 0.26, 0.11)	0.43
CERAD-Intrusions	− 0.65 (− 1.54, 0.24)	0.15	0.17 (− 0.04, 0.37)	0.12
Animal Fluency Score	0.92 (− 0.76, 2.60)	0.27	− 0.24 (− 0.58, 0.11)	0.17
Model 3: Model 2^B^ + Health and Health Behavior^C^	Cancer history *β* (95% CI)	*p*	Cancer history*aMed_High_vs_Low *β* (95% CI)	*p*
DSST Score	0.83 (− 1.86, 3.5)	0.53	− 2.37 (− 5.70, 0.96)	0.16
CERAD-WL	0.03 (− 0.76, 0.83)	0.94	0.45 (− 0.85, 1.74)	0.49
CERAD-DR	− 0.02 (− 0.45, 0.42)	0.93	− 0.05 (− 0.58, 0.49)	0.86
CERAD-Intrusions	− 0.22 (− 0.55, 0.10)	0.17	0.61 (0.07, 1.16)	0.03
Animal Fluency Score	0.23 (− 0.58, 1.05)	0.56	− 0.78 (− 2.01, 0.45)	0.203
Model 3: Model 2^B^ + Health and Health Behavior^C^	Cancer history *β* (95% CI)	*p*	Cancer history*aMed_HighestTertile_vs_Lowest Tertile *β* (95% CI)	*p*
DSST Score	− 0.16 (− 3.96, 3.65)	0.93	− 2.25 (− 6.59, 2.09)	0.30
CERAD-WL	− 0.09 (− 1.2, 1.02)	0.87	− 0.44 (− 2.15, 1.27)	0.61
CERAD-DR	− 0.39 (− 0.71, 0.63	0.91	− 0.38 (− 1.27, 0.50)	0.39
CERAD-Intrusions	− 0.16 (− 0.54, 0.22)	0.40	0.45 (− 0.32, 1.22)	0.24
Animal Fluency Score	− 0.18 (− 1.23, 0.87)	0.73	− 0.53 (− 1.95, 0.89)	0.45

Shown are estimates of the coefficients for the interaction terms of past cancer history with aMed as a continuous variable and with aMed as a binary variable for each of the five cognitive scores for the fully adjusted model

^A^Estimated using ordinary least squares regression, except for CERAD-Intrusions for which Poisson regression was used

^B^Model 1 (Cancer hx) + Demographics (age, race, gender, income, education)

^C^ Health and health behavior: diet, BMI, and smoking

**Table 4:** Association^A^ of past cancer history with cognition by age

Model 3: Model 2^B^ + Health and Health Behavior^C^	Cancer hx *β* (95% CI)	*p*	Cancer hx*(≥ 75 years vs. < 75 years) *β* (95% CI)	*p*
DSST Score	− 1.56 (− 3.84, 0.71)	0.17	2.95 (− 0.89, 6.79)	0.13
CERAD-WL	− 0.06 (− 0.72, 0.60)	0.85	0.77 (− 0.47, 2.00)	0.21
CERAD-DR	− 0.32 (− 0.72, 0.081)	0.11	0.80 (0.12, 1.47)	0.02
CERAD-Intrusions	0.05 (− 0.27, 0.37)	0.74	0.01 (− 0.50, 0.53)	0.96
Animal Fluency Score	− 0.48 (− 1.33, 0.37)	0.26	0.78 (− 0.74, 2.29)	0.31

Shown are estimates of the coefficients for the interaction terms of past cancer history with age as a binary variable for each of the five cognitive scores for the fully adjusted model

^A^Estimated using ordinary least squares regression, except for CERAD-Intrusions for which Poisson regression was used

^B^Model 1 (Cancer hx) + Demographics (age, race, gender, income, education)

^C^Health and health behavior: diet, BMI and smoking

The sensitivity analysis examining the effect of length of survivorship on cognition was performed with the fully adjusted model only and employed separate linear regression models for each measure of cognitive performance (Table 5).

**Table 5:** Association^A^ of time since first diagnosis with cognition

Model 3: Model 2^B^ + Health and Health Behavior^C^	Short-term survivors (< 5 years since diagnosis)		Long-term survivors (≥ 5 years since diagnosis)	
	Cancer history *β* (95% CI)	*p*	Cancer history *β* (95% CI)	*p*
DSST Score	− 2.66 (− 5.26, − 0.054)	0.046	0.067 (− 2.33, 2.46)	0.96
CERAD total	− 0.73 (− 1.85, 0.37)	0.18	0.49 (− 0.24, 1.22)	0.18
CERAD delayed	− 0.52 (− 1.04, − 0.004)	0.048	− 0.0038 (− 0.37, 0.37)	0.98
CERAD-Intrusions	0.05 (− 0.67, 0.77)	0.88	− 0.32 (− 0.64, 0.007)	0.055
Animal Fluency Score	− 0.58 (− 1.88, 0.71)	0.37	− 0.11 (− 1.023, 0.81)	0.81

Shown are estimates of the coefficients for the interaction term of binary aMed with survival time coded as 0 = non-survivor, 1 = short-term survivor, and 2 = long-term survivor

^A^Estimated using ordinary least squares regression, except for CERAD-Intrusions for which Poisson regression was used

^B^Model 1 (Cancer hx) + Demographics (age, race, gender, income, education)

^C^Health and health behavior: diet, BMI and smoking

We accounted for NHANES complex survey design of oversampling, clustering, and stratification by using the proper survey procedures. For linear regression models to test the association between cancer survivorship and cognition, we set alpha to 0.05. Alpha was set to 0.10 for interaction terms. All analyses were carried out with STATA version 17.0 (StataCorp LLC, College Station, TX, USA).

## Results

Table 1 shows the sample characteristics. The mean age was 68.9 years (standard error (SE): 0.2) with survivors being older than non-survivors. Survivors also had a higher prevalence of obtaining a 4-year college degree (35.6% vs. 29.3%, respectively) and a higher proportion of cancer survivors versus non-survivors were non-Hispanic White individuals (84.9% vs. 77.9%).

Table 2 shows the association between past cancer history and cognitive performance. In the unadjusted model (model 1), past cancer history was not associated with DSST Score (*β*: − 0.42; 95% (confidence interval (CI): − 2.95, 2.12), CERAD-WL (*β*: 0.10; 95% CI: − 0.31, 0.51), CERAD-DR (*β*: − 0.12; 95% CI: − 0.39, 0.15), CERAD-Intrusions (*β*: 0.06; 95% CI: − 0.14, 0.26), or Animal Fluency (*β*: − 0.07; 95% CI: − 0.75, 0.62).

In models that adjusted for demographic characteristics (model 2), the results were generally similar in magnitude and direction and were not statistically significant. Models additionally adjusted for health (model 3) tended to be the lowest in magnitude and were also not statistically significant.

**Fig. 1:**
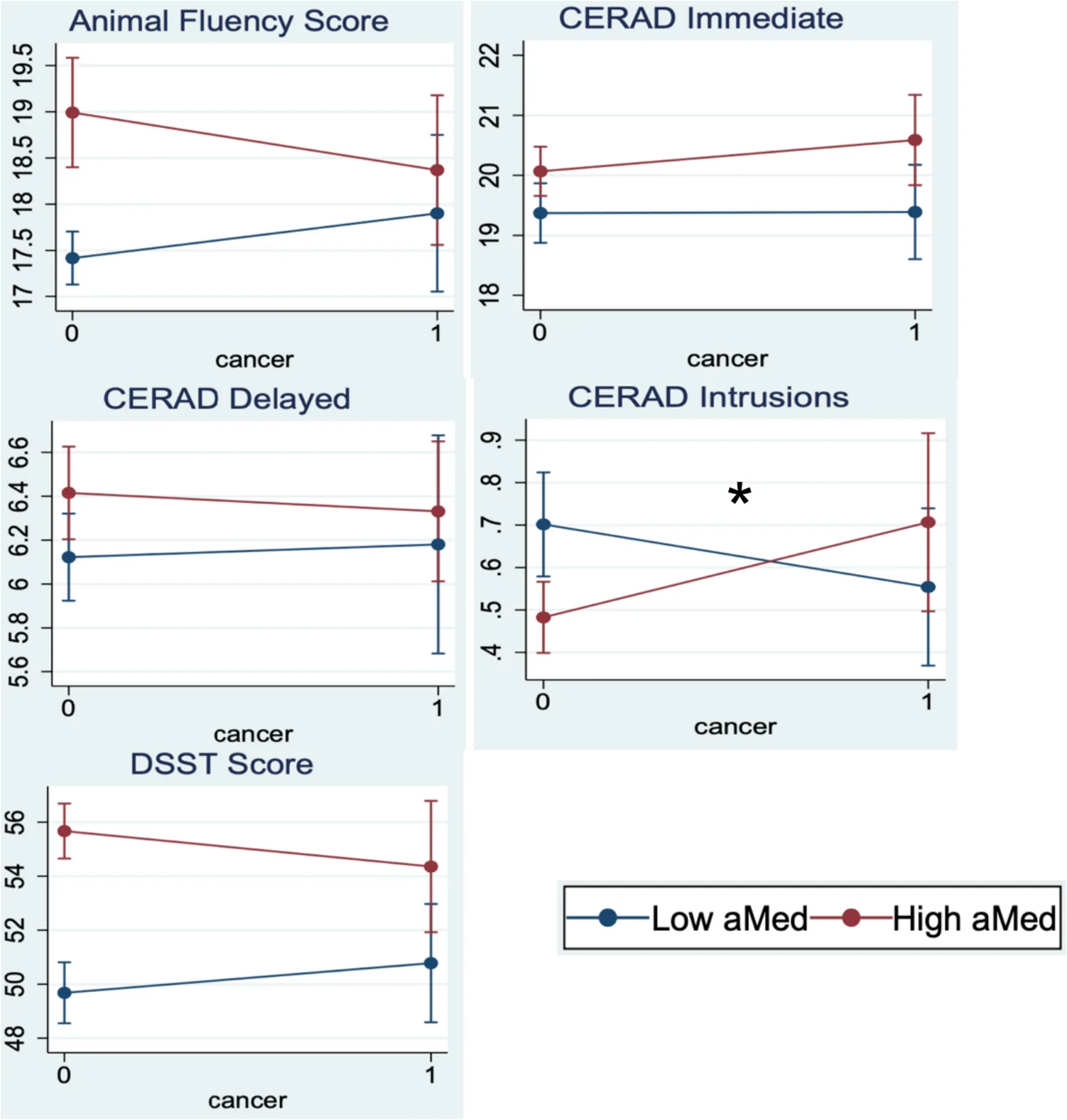
Shown are the scores for each cognitive test by cancer survivorship status (0 = control and 1 = survivor) and by aMed adherence (high or low). *p < 0.05

Table 3 shows whether the association between past cancer history and cognition varied by aMed as both a continuous and dichotomous variable (low vs. high Med Diet adherence). The association between past cancer history and cognition, as measured via CERAD-Intrusions, varied by aMed dichotomized at the median (Cancer history*aMed_High_vs_Low *β* [95% CI]: 0.61 [0.07, 1.16] at the alpha = 0.05 level. This means that the association between past cancer history and cognition was worse for high aMed adherers (versus low adherers), which contradicts our hypothesis that high Med Diet adherence could mitigate the potential adverse effects of cancer (and treatment) on cognitive impairment. Figure 1 illustrates these relationships.

We then examined what aspects specifically of the aMed score were driving the significant interactive effect for CERAD-intrusions and found, paradoxically, that fruit consumption above the median was associated with greater CERAD-intrusions, which is indicative of poorer cognition (Cancer history*MedFruit_High_vs_Low *β* [95% CI]: 0.74 [0.31, 1.17]).

While it was not the main exposure tested, continuous aMed score was positively and significantly (*p* < 0.05) associated with performance on every cognitive test for non-cancer controls. However, for cancer survivors, the association between performance and aMed score was statistically null for every cognitive test. This suggests a Med Diet may be more beneficial for non-cancer controls vs. survivors.

Table 4 presents results for the model with the Cancer history*age interaction. The association between past cancer history and cognition, as measured by CERAD-DR, varied by age (*β*: 0.80; 95% CI: 0.12, 1.47), and the total effect of Cancer history on cognition for those at higher age was positive (*β*: 0.50; 95% CI: 0.053, 0.95). At a lower age, the effect of cancer history was null (*β*: − 0.31; 95% CI: − 0.69, 0.078). This means past cancer history has a positive relationship with CERAD-DR in those ≥ 75 years of age but no relationship with CERAD-DR in those < 75 years of age.

### Sensitivity analysis

We next performed a sensitivity analysis to determine if time since diagnosis may be influencing the finding that cognition is not associated with past cancer history. To do so, we replaced the cancer history variable with a categorical one that also included length of survival (i.e., 0 = non-survivor, 1 = short-term survivor, and 2 = long-term survivor). For short-term survivors, cancer history was negatively associated with DSST Score (*β*: − 2.66, 95% CI: − 5.26, − 0.054) and CERAD delayed (*β*: − 0.52, 95% CI: − 1.04, − 0.004). For long-term survivors, there was no effect of cancer history on cognition.

## Discussion

Using NHANES data from the 2011–2012 and 2013–2014, we show that past cancer history is not significantly associated with cognitive performance after adjusting for several potential confounders, including age and education. This differs from our hypothesis that cancer survivors would have poorer cognitive performance compared to non-cancer controls. These results also differ from prior studies showing cancer survivors have poorer cognitive performance [28]. For example, Williams and colleagues used NHANES data from 1999 to 2002 and reported that cancer survivors have worse performance on DSST compared to non-cancer controls [52]. The difference between the results of our study and that of Williams et al. may be attributable to reduced cancer-related mortality that occurred continuously from the early 1990’s to 2011 [41]. Reductions in cancer mortality have occurred due to advances in cancer prevention, early detection, and treatment [41], suggesting that cancers are being diagnosed at earlier stages, resulting in less aggressive treatments. In turn, this may result in less CRCI over time and less of an effect of cancer in our study compared to earlier ones, such as Williams et al. Lastly, these prior studies also include some survivors within 5 years of diagnosis, whereas NHANES includes some survivors 10 + years post-diagnosis who may have recovered from CRCI. Indeed, we found that short-term survivors exhibited lower scores on CERAD delayed and DSST compared to long-term survivors. These factors may have contributed to our null findings.

Another explanation for the difference between the results of our study and those of Williams et al. is that the cancer survivors in our sample were younger and were more educated. As younger age and higher education levels are strongly correlated with higher cognitive performance [29], this may have mitigated any potential cognitive deficit in survivors relative to controls, or at the very least, any difference was not precisely measured by the NHANES neuropsychological evaluations, which may lack the sensitivity required to detect the often-subtle impairments of CRCI (Ahles et al., 2018). While we did control for age and education in our models, our sample size may have been too small to detect effects between cancer survivors and non-cancer controls. It should be noted, however, that a null association between past cancer history and cognitive performance in cross-sectional studies that include older adults and all types of cancer and treatment has previously been reported [25, 39]. This may be because not all cancer treatments, e.g., non-CNS radiation, result in cognitive impairment [26]. Thus, our results may have been affected by the inclusion of survivors who would not generally be expected to have cognitive impairment from cancer treatment. Moreover, individuals with poor cognition were excluded from the cognitive testing of NHANES. This may have excluded cancer survivors suffering from CRCI and also may have led to our generally null results.

Furthermore, the majority of studies of CRCI have been conducted in adults in their 50 s [28, 50]. The mean age of survivors in the current study is nearly 71 years, and therefore, the survivors in this study may exhibit a selective survival effect whereby having survived cancer and having lived to an older age may result from superior overall health, including cognitive health, compared to a non-cancer control of a similar age. Indeed, cancer survivors 75 years and older in this study appear to have higher CERAD-DR scores than survivors younger than 75 years. This cognitive survival effect has been shown in other populations, such as in older, African American adults: in the Baltimore Study of Black Aging, African American adults older than 63 had no decline in inductive reasoning whereas those younger than 63 did (Tan, et al., 2021). While such reasoning suggests a similar phenomenon would occur in William et al. (2016) where the survivors were even older than those in the current study, this was not the case. This could be due to several factors, including demographic, socioeconomic, and/or environmental differences between those tested in 1999–2002 and those tested in 2011–2014.

Our analysis also tested for effect modification by aMed and found that the association of past cancer history with cognition varied based on aMed adherence (as a binary variable). Specifically, the association between past cancer history and cognitive performance was worse for high adherers relative to low adherers in terms of CERAD-Intrusions. This was unexpected and differs from our hypothesis that any objective cognitive deficit associated with past cancer history would be lower among those with higher (versus lower) adherence to a Med Diet. This finding diverges from some prior literature. In a 26-week randomized controlled clinical trial, among 143 early-stage breast cancer survivors undergoing chemotherapy and radiation, participation in a diet and physical activity intervention increased cognitive flexibility significantly more compared to a usual care group [7]. In a cross-sectional study among breast cancer survivors (post-treatment) and non-survivors, vegetable and/or fruit consumption was positively associated with executive functioning [54].

The differences between the results of our study and Carayol et al. and Zuniga et al. may stem from the fact that the cohorts in these two studies were younger than our cohort. The participants in Zuniga et al. had a mean age of 56 years and those of Carayol et al. 52 years, both much younger than the survivors in the current study (mean age 71 years). There is evidence that cancer and its treatment accelerates aging [19], placing cancer survivors at higher risk for the conditions of older age, such as cachexia, sarcopenia, and associated unintentional weight loss (Kleckner et al., 2022), compared to the controls in our study. High-protein meals and snacks treat unintentional weight loss, which is related to cognitive decline (Kleckner et al. [2]). However, plant-based meals, such as those more common in a Mediterranean Diet, have been shown to have a third less protein than typical animal-based meals [32]. Thus, cancer survivors in our sample adhering to a Med Diet may have had too low of protein consumption to combat their cancer-related accelerated aging and associated symptoms, potentially leading to more limited benefits of the Mediterranean Diet on cognitive performance and a more negative effect of cancer on cognition. However, among survivors, there was no significant difference in protein intake per 1000 kcal between high and low Med Diet adherence.

Moreover, the above studies and, to our knowledge, all other studies investigating cognition and diet in cancer survivors have been in breast cancer survivors. NHANES includes all cancer types and treatments, which may have led to our results conflicting with prior studies regarding cancer and cognition. It would be beneficial to investigate the interaction of a Med Diet with cancer survivorship among individuals with different cancer types and treatments. A prospective study that measures diet and cognition at diagnosis and at various time points post-treatment could detect these potentially time-varying associations between diet and cognition.

Past cancer history also varied by age, whereby the association of cancer survivorship with CERAD-DR was higher at a higher age (≥ 75 years) compared to a lower age (< 75 years). In a recent analysis of the Health and Retirement Study cohort, cancer survivors had significantly higher memory scores than non-cancer controls *before* diagnosis, suffered a loss in memory capacity immediately after diagnosis that then equalized their memory with that of non-survivors, but over the next decade maintained a slower rate of memory decline after diagnosis compared to non-cancer controls [36]. Cancer survivors in this NHANES cohort may have had a similar memory trajectory, and this may account for the inability to detect memory differences between survivors and non-cancer controls until older age.

The strengths of this study include the use of a large, nationally representative dataset. It is also the first to report CERAD and Animal Fluency scores for cancer survivors using NHANES data. Our study, however, is not without limitations. Because the data is cross-sectional, causality cannot be determined between diet and cognition nor between cancer and cognition. Given the myriad of variables associated with both cognition and cancer survivorship that were not measured by NHANES, variables such as post-traumatic stress syndrome and fatigue, residual confounding cannot be ruled out. Secondly, we were unable to provide estimates for different cancers and treatments as treatment data were not collected, and not enough survivors of any cancer were available to conduct a meaningful statistical analysis. Third, our estimates when stratifying our sample by length of survivorship (i.e., time since diagnosis) are not generalizable to anyone 80 years and older because NHANES right censors at 80 years of age, making it impossible to identify the length of survivorship of anyone 80 years and older. The proportion of cancer survivors 80 years and older is 19% in this dataset, representing a considerable amount of missing data in our analysis pertaining to length of survivorship. Future larger studies that do not right censor on age would be able to identify better which cancers, cancer treatments, and lengths of survivorship are related to cognitive impairment and how diet interacts with these relationships. Fourth, type 1 error, or false positives, may also play a role as we made several comparisons. Only one subscore of the aMed score, fruits, interacted negatively with cancer survivorship, suggesting that this finding was coincidental. Lastly, if cancer history is associated with impaired cognition, individuals with cancer may therefore mistakenly misreport their medical history, thus leading to cancer survivors potentially classifying themselves as non-cancer controls. This may have led to the appearance of poorer cognition among controls, thus mitigating any CRCI effect that existed. Likewise, CRCI may have also led to misreporting of diet, leading to more random errors and less precise dietary estimates among cancer survivors.

In conclusion, this study is the first, to our knowledge, to use NHANES data from 2011 to 2014 to examine the association between past cancer history and cognitive performance. We show that past cancer history is significantly and negatively associated with cognitive performance but only among short-term survivors using a subset of available data from NHANES. When including all cancer survivors, and after controlling for several confounders, we did not observe a relationship between past cancer history and cognition. However, the association did vary by Med Diet adherence and age, in an unexpected manner. Specifically, the association of past cancer history with cognitive performance was lower at higher (versus lower) levels of Med Diet adherence, and the association of past cancer history with CERAD-DR was higher at a higher age (≥ 75 years) compared to a lower age (< 75 years). Given these unexpected results and the use of a highly heterogenous population in terms of cancer type, larger, prospective studies in older cancer survivors are needed.

## Data Availability

The data and data analysis scripts underlying this article are available in Box, at https://uofi.box.com/s/tjc8yop079y3u11umj1kzv3jv9gki4hf. The datasets were derived from sources in the public domain: NHANES at https://wwwn.cdc.gov/nchs/nhanes/continuousnhanes/default.aspx?BeginYear=2011 and https://wwwn.cdc.gov/nchs/nhanes/continuousnhanes/default.aspx?BeginYear=2013.
